# The Influence of Place Attachment on Pro-Environmental Behaviors: The Moderating Effect of Social Media

**DOI:** 10.3390/ijerph16245100

**Published:** 2019-12-13

**Authors:** Jian Xu, Ruixia Han

**Affiliations:** 1 School of Media and Communication, Shanghai Jiao Tong University, 800 Dongchuan RD, Minhang District, Shanghai 200240, China; xujian@sjtu.edu.cn; 2 China Institute for Urban Governance, Shanghai Jiao Tong University, 1954 Huashan RD, Xuhui District, Shanghai 200052, China; 3 Institute of Cultural Innovation and Youth Development, Shanghai Jiao Tong University, 800 Dongchuan RD, Minhang District, Shanghai 200240, China

**Keywords:** social media, place attachment, pro-environmental behavior, moderating effect, environmental governance

## Abstract

China is facing tremendous pressure to improve the environment. How to promote pro-environmental behaviors at the individual level is an important research topic. This study examines the relationship between social media usage, place attachment, and pro-environmental behavior based on a survey of 550 Chinese citizens. The results show that: (1) Place attachment and social media usage for environmental information acquisition have positive correlations with pro-environmental behaviors; (2) social media usage for environmental information acquisition moderates the relationship between place attachment and pro-environmental behaviors. Our survey also finds that social media play a more important role than traditional media in influencing pro-environmental behaviors. Our findings indicate that social media is changing the traditional relationship between place attachment and pro-environmental behavior. We should pay more attention to this positive role of social media and encourage citizens’ pro-environmental behavior.

## 1. Introduction

According to the data published by NASA’s Earth Observing System Data and Information System, in the 0–100 scoring system, China scores 50.74 in Environmental Performance Index, 31.72 in Environmental Health, and 63.42 in Ecosystem Vitality. The overall EPI ranking is 120 in all 180 countries, ranking in the middle and rear positions [1]. This indicates an urgency and necessity of environmental protection in China. The Chinese government is also aware of this problem and has launched the “Beautiful China” program, which aims to promote various environmental protection actions. For example, Shanghai has launched a strict standard garbage sorting campaign this year. However, environmental protection is first and foremost a personal behavior. Plans for environmental protection can only succeed with the cooperation from individuals and their pro-environmental actions.

Many scholars have researched the influencing factors of Chinese citizens’ public behavior. Their research can be basically classified into the following four theoretical frameworks: the reasoned action theory [2], the planned behavior theory [3], the norm activation mode [4], and the value-belief-norm theory (VBN) [5], which is developed from the normative activation theory by Stern. However, the above theoretical frameworks mainly focus on the normative factor of pro-environmental behaviors, regardless of whether they are externally formed or internalized in personal values. They mainly concern the cognitive (e.g., environmental knowledge, cognitive biases) and behavioral (e.g., community membership, perceived behavior control) aspects, but no sufficient attention is paid to the emotional interaction between people and the environment. Therefore, some scholars advocate a rational integration model of pro-environmental behaviors by adding in the dimension of emotional involvement [6]. In fact, in recent years, scholars have examined the influence of place attachment on pro-environmental behaviors. For example, Halpenny [7], and Song et al. [8] have analyzed the effects of different dimensions of place attachment on pro-environmental behaviors. These studies further demonstrate that place attachment does affect individual pro-environmental behaviors.

One fact we tend to ignore is that in an Internet society, we live both in the physical/real space and the virtual online space. With the development of social media, traditional local community is changing. Social media is changing the way we connect with places. In China, the most widely used WeChat social platform has a penetration rate of 97% among Chinese netizens [9]. Video social media, represented by TikTok, have also grown rapidly and reformed the way Chinese using the social media. Does social media usage promote the pro-environmental behaviors of the Chinese public? How effective are they compared to traditional media? Do social media change the way in which place attachment influences pro-environmental behaviors? With these questions in mind, we conduct an empirical survey of the Chinese public. We try to identify key factors affecting the Chinese public’s pro-environmental behaviors and the role of social media in changing the relations between place attachment and pro-environmental behavior.

## 2. Research Background and Hypothesis

### 2.1. Place Attachment (PA) and Pro-Environmental Behavior (PEB)

Place attachment refers to people’s emotional link to their place of residence [10]. In the concept of place attachment, emotion is considered to be the core element in the relationship between people and place. Although some scholars, such as Scannell et al. [11] point out that the concept of place attachment should also include cognitive and behavioral factors, emotion is still the core element. Regarding the compositional dimensions of place attachment, different scholars give multiple answers. For example, Williams and Jerry [12] believe that place dependence and place identity should be included, while Kyle et al. [13] contend that place identity, place dependence, and social bonding should be included. Hammitt et al. [14] insist that there are five dimensions which make up place attachment: place familiarity, belongingness, identity, dependence, and rootedness. Regardless of the scholars’ disagreement in the number of dimensions, they all agree that place attachment is an effective concept for describing the human–land relationship. It is precisely due to the place attachment that people tend to behave in a pro-environment manner.

Pro-environmental behaviors refer to the daily behaviors that are beneficial to the environment or help protect the environment [15]. Pro-environmental behaviors are significantly related to climate change, environmental pollution, and natural disasters, because the more clearly people recognize the pressure of environmental damage, the more likely they are to conduct environmental protection activities. This mode of correlation is called the cognitive stress model of pro-environmental behaviors [16]. In fact, emotions may also contribute to pro-environmental behaviors. For example, Coelho et al. [17] find that positive emotions can promote pro-environmental behaviors by mediating environmental concern, while negative emotions directly affect pro-environmental behaviors. Place attachment is a positive emotional connection between people and place. This kind of emotional connection will also lead to pro-environmental behaviors.

A large number of studies have also confirmed the positive impact of place attachment in promoting pro-environmental behaviors. For example, Scannell and Robert [11] argue that civic place attachment, as one form of place attachment, can effectively help predict citizens’ pro-environmental behaviors. Halpenny [7] found that place identity moderates the relationship between place dependence and pro-environmental behaviors. Dividing place attachment into four aspects: place dependence, place identity, place affect, and place social bonding, Ramkissoon et al. [18] found that place satisfaction moderates the relationship between these four aspects of place attachment and pro-environmental behavioral intentions. Zhang et al. [19] discovered that place attachment is more likely to affect pro-environmental behavior than the consciousness of disaster or values. Based on the summary of a large number of studies, Carrus et al. [20] pointed out that place attachment includes social and physical dimensions of human–land emotional relationship. Both environmental psychology and sociological researches prove that place attachment is closely related to pro-environmental behavior, where community identity plays an important role. All of these studies have shown that place attachment could promote pro-environmental behaviors. So we assume that:

**Hypothesis 1 (H1).**  *Place attachment has a positive correlation with pro-environmental behaviors.*

### 2.2. Social Media (SM) and Pro-Environmental Behavior (PEB)

The relationship between media usage and pro-environmental behavior has also attracted much scholarly attention. Chan [21] conducted a survey of 173 households in Hong Kong and found that mass media can affect residents’ pro-environmental behavior by influencing their subjective identification of social norms. Chan’s research effectively combines the study of mass media’s influence on individual attitudes and the planned behavior theory’s analysis model of pro-environmental behavior. Subsequent researches further enrich and expand the model. For example, Lee [22] categorizes media exposure, social exposure, and biospheric value orientation into an attitude-intention-behavior model of pro-environmental behavior. Lee demonstrates that media exposure does have a significant impact on adolescents’ pro-environmental behavior. Huang’s study on Taiwanese residents [23] found that global warming information obtained by individuals through media (mainly including television, newspapers, and the Internet) affect their pro-environmental behavior. Trivedi et al. [24] discovered that media positively affects the environmental concerns and negatively influences the internal environmental attitudes, and ultimately affects the green buying behavior. Ho et al. [25] combined the theory of planned behavior with the theory of media dependence and found that, traditional media attention and interpersonal communication (interpersonal discussion with friends, family, and colleagues about environmental issues) moderate the influence of media dependence on green purchasing behavior. These studies demonstrate that there is a correlation between media usage and pro-environmental behavior, but whether social media usage and environmental behavior are related needs to be further explored.

Niki and Wilson [26] proved that social media effectively activates people’s awareness of interpersonal comparison (compare with others in pro-environmental behavior) and promote their pro-environmental behavior by improving their cognition of norms. Hynes also summarizes in detail the paths by which social media influences people’s pro-environmental behaviors. For instance, the display function of social media can make people’s daily behaviors in some small environmental protection activities have a magnifying effect [27], which in turn convince and influence the effects of other public behaviors [28]. Social media has a strong social comparison function, which persuades people to change their behavior [29,30,31]. Social media makes it easier for people to see the results of green behavior, thereby motivating people to engage in pro-environmental behaviors [32,33]. For example, social media’s recording function allows people to have an intuitive feeling of their own energy-saving and environmental performance, thus promoting their pro-environmental behaviors [31,34]. In summary, the social display and social record functions of social media, the stimulation of people’s social comparison psychology, and the improvement of self-efficacy of environmental protection all contribute to the promotion of pro-environmental behavior. Therefore, we introduce the following assumption.

**Hypothesis 2 (H2).**  *Social media has a positive correlation with pro-environmental behavior.*

### 2.3. Social Media (SM), Place Attachment (PA) and Pro-Environmental Behavior (PEB)

The above studies show that place attachment promotes people’s pro-environmental behaviors, and that people’s positive attachment to their place will encourage their pro-environmental behaviors. Carrus et al. [35] believe that this association can even be traced back to the parent–child relationship. To a certain extent, place attachment is the expansion of parent–child attachment, and this attachment will encourage people to make positive behaviors. The opinion of Feldman [36] also indirectly indicates that pro-environmental behavior itself is a constituent dimension of people’s expression of place attachment. At the same time, place attachment is also closely related to community identity, and community will bring pressure to promote positive environmental behavior. The relationship between place attachment and pro-environmental behavior has been confirmed in various studies.

Like traditional media, social media initiate their influence on pro-environmental behavior by affecting people’s attitudes and behaviors. But unlike traditional media, social media is much more powerful in combining mass communication and interpersonal communication. Ho et al. [25] found that interpersonal communication, like mass media, is an important variable that affects people’s pro-environmental behavior. Interpersonal communication and mass media together affect people’s attitudes and their green purchase behavior. The immediacy, sociality, and social comparison characteristics of social media combine media pressure and group pressure, promote people’s pro-environmental behavior.

Although various studies and theories have led us to believe that social media and place attachment affect pro-environmental behavior, no research focuses directly on the relationship between them. However, social media’s combination of the characteristics of both interpersonal and media communications as well as their close association with local communities gives us reason to believe that certain form of correlation does exist between social media, place attachment, and environmental behavior. First, as Manuel Castells [37] describes cyberspace is reshaping our understanding of time and space, and it recreates local spaces. In other words, the Internet provides the possibility for local people to gather online, and social media improves the efficiency of aggregation. Second, Scannell and Gifford [11] proposed the Person-Process-Place (PPP) model of place attachment, which is different from the traditional man-land connection. It suggests that place attachment is a multi-level concept which consists of three major structures: people, psychological processes, and places. The mediating role of psychological processes provides a broad space for social media to act. Social media can promote the interaction of local people, further strengthen their attachment to the same place, and ultimately promote their feedback-pro-environmental behavior, so we have reason to speculate:

**Hypothesis 3 (H3).**  *Social media moderates the relationship between place attachment and pro-environmental behavior.*

### 2.4. Social Media (SM),Traditional Media (TM), and Pro-Environmental Behavior (PEB)

Kara Chan [21] discovered that mass media, as the main source of subjective norms, significantly affects people’s pro-environmental behaviors. Mileti [38] demonstrates that media communication plays a mediating role between risk perception and pro-environmental behavior. Many studies have shown that media magnify people’s perception of environmental risk, which in turn affects people’s pro-environmental behaviors. Zeng et al. [39] believes that new media is more capable of amplifying people’s perception of environmental risks. Relying on the cultivation theory and the use and gratifications theory, Holbert et al. [40] pointed out that watching TV enhances people’s concern for the environment and thus affects their pro-environmental behavior. When we approach the issue of media’s impact on pro-environmental behaviors, most direct empirical experiences tell us that media promotes pro-environmental behaviors by disseminating knowledge and information of environmental protection. For example, Brothers [41] demonstrated that television reports on environmental issues help improve the public’s environmental knowledge. Zhao [42] also discovered that media usage mediated the effects of age, race, and education on perceived knowledge about global warming. However, the influence of media on pro-environmental behavior discussed above is mainly based on traditional media types. For example, Holbert’s research [40] mainly focused on television, and Huang’s research [23] included television, newspapers, and the Internet, Ho.’s study [25] categorizes newspapers and television as traditional media compared with the Internet, analyzing their relationship with pro-environmental behavior, but there is little research into social media. In fact, as analyzed in the previous article, social media can play a greater role in pro-environmental behavior because of its social comparative function [29] and social display function [27] etc., which can promote people’s normative perception [26] and self-efficacy [31]. It should be noted that, as the above research shows, when analyzing the relationship between media and pro-environmental behaviors, we can find that the types of media are expanding. In the last 40 years, people have often compared the Internet as a new medium with other media. However, after the emergence of social media, it has been compared with other media, including the Internet, because it combines the characteristics of interpersonal and media communication. The previous forms of Internet use, represented by portals, have been classified into the traditional media category. We have reason to believe that social media is playing a different role in promoting pro-environmental behavior than other types of media, including the Internet. So we speculate that:

**Hypothesis 4 (H4).**  *The correlation between traditional media and pro-environmental behavior is significantly lower than that between social media and pro-environmental behavior.*

**Figure 1:**
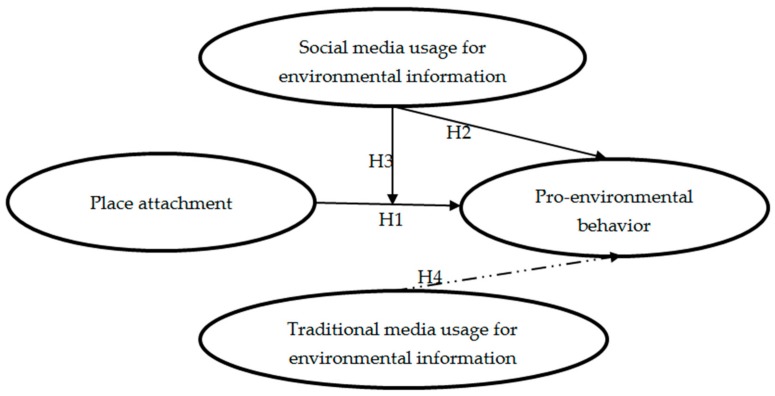
A proposed core model depicting the process of pro-environmental behavior.

Summarizing the above research concerns, we use the following figure (Figure 1) to express the relationship between core assumptions.

## 3. Research Design

### 3.1. Sample and Data Collection

Our survey collected data online. Our data come from the online questionnaire platform “https://www.wjx.cn/” in mainland China. We select our volunteers randomly from the 2.6 million registered users of the platform. By setting IP address, computer, mobile phone, user limit, and logic problems, we want to ensure that each sample only fill out the questionnaire once. We finally collected 550 valid questionnaires (Table 1). Our investigation lasted seven days (20 August 2019—27 August 2019). Covering residents from all the 30 provincial administrative divisions in mainland China except for Xinjiang, our samples are representative enough to reflect the basic condition of Chinese public’s pro-environmental behaviors and attitudes. Considering the actual gender ratio of China’s population at 51:49, we weighted the female data in the sample to achieve a match with the overall gender ratio of the population. In addition, because this sample is mainly from the urban population, the proportion of university graduates in the working stage is relatively high.

### 3.2. Measurement

#### 3.2.1. Pro-Environmental Behavior (PEB)

The public’s pro-environmental behavior is the dependent variable in our study. How to measure pro-environmental behavior is an important issue for this study. There have been various measurements of pro-environmental behavior in the international academic circle, such as Bratt (1999) [43], Gatersleben et al. (2002) [44], Dono et al. (2010) [45], Davis et al. (2011) [46], and Tobler et al. (2012) [47]. Markle launched his own measurement scale in 2013 by synthesizing the above-mentioned studies [48]. They make the measurement of pro-environmental behavior more specific and precise. However, it should be noted that the measurement of pro-environmental behavior should also take into account the specific socio-cultural environments. Based on the summary of relevant measurements in the international academic community, the Chinese scholar Hong developed new measurement scales and applied them to the Chinese General Social Survey (CGSS) in 2003, 2010, and 2015. Considering the importance of the comparative studies, this scale was used in this study. The corresponding question in the questionnaire is: “Have you engaged in the following activities or behaviors in the past year?” Specific actions include: (1) recycling; (2) discussing environmental issues with relatives and friends; (3) bringing a shopping basket or shopping bag when grocery shopping; (4) reusing plastic bags; (5) donation for environmental protection; (6) actively paying attention to various environment-related information; (7) actively participating in environmental publicity and education activities organized by the government and your institution; (8) actively participating in environmental protection activities organized by private environmental protection groups; (9) maintaining forests or green spaces at your own expense; (10) actively participating in environmental rights activities. We give our correspondents the following selections: 0 = never; 1 = occasional; 2 = frequently, 3 = always. The internal consistency coefficient of the scale is 0.741 (Cronbach’s Alpha), and the total value of each variable is between the range of 0–30.

#### 3.2.2. Place Attachment (PA)

There have been a large number of operational scales for the study of place attachment [12,49,50]. Because our study focuses on pro-environmental behavior, we correspondingly select the natural place attachment scale developed from the study of Scannell and Gifford [18]. The main questions included in the scale are: (1) I feel connected to the community; (2) I am attached to the city; (3)When I am away I miss the community; (4) I am proud of my city; (5) This city is special to me; (6) I respect what this city stands for; (7) People like me living here; (8) This community reflects who I am; (9) The green spaces here are special; (10) I am attached to the green spaces here; (11) The natural environment is special to me. In the data file, 1–5 represents an increase in consent. The internal consistency coefficient of the scale is 0.855 (Cronbach’s Alpha), and the total value of each variable is in the range of 0–55.

#### 3.2.3. Social Media Usage for Environmental Information Acquisition (SME)

Considering the special Chinese context of social media usage, the types of social media we measure include WeChat, Weibo, Tik-tok, Kwai, QQ, BaiduTieba, Zhihu, Douban, Facebook, Twitter, and Instagram. The measurement of environmental information acquisition mainly includes four categories (1) information on environmental change; (2) various discussions on the environment; (3) how other people and places deal with environmental issues, and (4) ways and means to protect the environment. We assign one point for each category. We cross-form a matrix of social media types and environmental information acquisitions, examine the distribution of each respondent on each crossover option, and ultimately form a score. The final scoring weighted average range is 0–4. The internal consistency coefficient of the scale is 0.807 (Cronbach’s Alpha).

#### 3.2.4. Traditional Media Usage for Environment Information Acquisition (TME)

Traditional media include newspaper, magazine, radio, television, and the Internet. It should be particularly noted that, in order to make the respondents clear the difference between the Internet and social media, the Internet special description here refers to Internet sites other than social media, which are represented by portal sites. We use the same subject matter to measure the function of traditional media, as we did to social media. One point is assigned for each type. The final scoring weighted average range is 0–4. The internal consistency coefficient of the scale is 0.777 (Cronbach’s Alpha).

#### 3.2.5. Control Variables

Demographic variables are measured as follows: gender: 1 = male, 0 = female, and female as control. Age: Calculated using the year 2019 minus the year of birth. Education: 1 = junior high school and below, 2 = high school/secondary school, technical school, 3 = college, university, 4 = master, 5 = doctor and above. Income per year: 1 = Less than 10,000 rmb, 2 = 10,000–30,000 rmb, 3 = 30,000–50,000 rmb, 4 = 50,000–100,000 rmb, 5 = 100,000–200,000 rmb, 6 = 200,000–500,000 rmb, 7 = 500,000 rmb or more. Community participation includes: (1) churches, religious groups, (2) sports and fitness groups, (3) cultural and educational groups, (4) professional associations (such as educational associations, business associations), and (5) school-related groups (Alumni Association), (6) Owners’ Committee, (7) Clan Association, Family Association, Association, 1 point for each participation, ranging from 0–7, the internal consistency coefficient of the scale is 0.754 (Cronbach’s Alpha).

##### Environmental Knowledge (EK)

Our questionnaire designs ten questions to assess the environmental knowledge of our correspondents which has been tested by Hong [51]. They include: (1) Car exhaust will not pose a threat to human health; (2) excessive use of chemical fertilizers and pesticides will lead to environmental damage; (3) the use of phosphorus-containing detergent will not cause water pollution; (4) fluorine emissions from fluorine-containing refrigerators will become a factor that destroys the ozone layer; (5) the production of acid rain has nothing to do with burning coal; (6) Species are interdependent on each other, and the disappearance of one species will have a chain reaction; (7) in the air quality report, the third-class air quality means better than the first-class air quality; (8) single-species forest is more likely to cause pests and diseases; (9) in the water pollution report, Class V means better water quality than Class I; (10) the increase of carbon dioxide will become a factor of climate warming. Among them, questions 1, 3, 5, 7, and 9 are incorrect expressions, and questions 2, 4, 6, 8, and 10 are correct expressions. After 1, 3, 5, 7, and 9 are reverse coded for further analysis, we get an integrated variable with a range of 0–10. The internal consistency coefficient of the variable is 0.773 (Cronbach’s Alpha).

##### Environmental Concerns (EC)

We measure the environmental concerns by using the China version of the New Ecological Paradigm (NEP) scale, which also has been tested by Hong [52]. Our questions include: (1) We are approaching the limit of the number of people the earth can support. (2) When humans interfere with nature it often produces disastrous consequences. (3) Humans are seriously abusing the environment. (4) Plants and animals have as much right as humans to exist. (5) The balance of nature is strong enough to cope with the impacts of modern industrial nations. (6) Despite our special abilities, humans are still subject to the laws of nature. (7) The so-called “ecological crisis” facing humankind has been greatly exaggerated. (8) The earth is like a spaceship with very limited room and resources. (9) The balance of nature is very delicate and easily upset. (10) If things continue on their present course, we will soon experience a major ecological catastrophe. The corresponding options in the data file are: 2 = strongly agree; 1 = agree; 0 = average, −1 = disagree, −2 = strongly disagree. 5 and 7 are reverse coded for further analysis, the scale ranges from −22 to 22, and the internal consistency coefficient is 0.714 (Cronbach’s Alpha).

##### Environmental Risk Perception (ERP)

We measure the environmental risk perception by asking the following question: “How severe are the following types of environmental pollution in your area?” Specific environmental issues include: (1) air pollution; (2) water pollution; (3) noise pollution; (4) industrial waste pollution; (5) domestic garbage pollution; (6) lack of green space; (7) destruction of forest vegetation; (8) deterioration of cultivated land quality; (9) shortage of fresh water resources; (10) food pollution; (11) desertification; (12) reduction of wildlife. The corresponding options in the data file are: 1 = very light; 2 = light; 3 = average, 4 = heavy, 5 = very heavy. The variable takes a range of 0–5 by weighted averaging. The internal consistency coefficient is 0.818 (Cronbach’s Alpha).

### 3.3. Statistical Analysis

We conducted ordinary least squares hierarchical regression analysis for hypothesis testing in SPSS 19.0 (SPSS Inc., Chicago, IL, USA). Specifically, the pro-environmental behavior is set as the dependent variable, and other variables as predictor variables are put into the regression model in two steps. In the first step, we put two groups of variables into the models. They are control variables and two types of media composure variables (TME and SME), then we get the models of 1E2 and 4 which were combined to test H4. In the second step, first we put place attachment into the regression model to get model 3 for testing H1. Second, we put SME to get model 4 for testing H2. At last, we put PA and SME to get model 5, and put interaction variables (PA × SME) into the regression model to get model 6. We combined models 5 and 6 to test H3.

## 4. Results

### 4.1. Descriptive Statistics

With a Pearson correlation test on variables in groups of two, we are able to show our findings in Table 2: on the personal level, age (r = 0.152, *p* < 0.01), income (r = 0.172, *p* < 0.01), and community participation (r = 0.409, *p* < 0.01) are significantly positively correlated with pro-environmental behavior. In other words, people who are older, have a higher income or more actively participate in community activities are more likely to engage in pro-environmental behavior. Environmental knowledge (r = −0.11, *p* < 0.05) and environmental risk perception (r = 0.152, *p* < 0.01) also have a significant correlation, so do place attachment (r = 0.235, *p* < 0.01) and social media usage for environment information acquisition (r = 0.289, *p* < 0.01). People with stronger environmental risk perception and place attachment are more engaged in pro-environmental behavior. However, people with more environmental knowledge are less engaged in pro-environmental behavior, which is consistent with other data already available in China. It is worth noting that neither environmental concerns nor traditional media usage for environment information acquisition has a significant correlation with pro-environmental behavior.

### 4.2. Hypothesis Testing

After standardizing the data, we used a multi-level regression method to introduce variables in steps to test the four hypotheses. All results are shown in Table 3.

First, we use control variables as independent variables to form a reference model (Model 1). Second, we introduce traditional media usage for environmental information acquisition (TME) into Model 2 for H4 testing. Third, we introduce place attachment (PA) into Model 3 for H1 testing and introduce social media usage for environmental information acquisition (SME) into Model 4 for H2 and H4 testing. Lastly, Models 5 and 6 are prepared for testing H3. The test results are as follows:

Model 3 introduces place attachment (PA) as an independent variable into the regression model, and we can find that the predictive effect of place attachment on the pro-environmental behavior is significant (β = 0.123, *p* < 0.001), thus proving H1. That is to say that place attachment has a positive correlation with pro-environmental behaviors.

Model 4 introduces social media usage for environmental information acquisition (SME) as an independent variable into the regression model, and it can be found that social media usage for environmental information acquisition (SME) has a significant effect on predicting pro-environmental behavior (β = 1.242, *p* < 0.001), thus proving H2.That is to say that social media has a positive correlation with pro-environmental behaviors.

Model 5 uses both place attachment (PA) (β = 0.114, *p* < 0.001) and social media usage for environmental information acquisition (SME) (β = 0.119, *p* < 0.001) as independent variables, and finds that both have a significant effect on pro-environmental behavior. Model 6 introduces the interaction effects (PA × SME) of place attachment (PA) and social media usage for environmental information acquisition (SME) as variables, and finds that the effect of place attachment (PA) (β= 0.051, *p* > 0.05) is no longer significant, whereas social media usage for environmental information acquisition (SME) (β = 2.730, *p* < 0.01) and its interaction with place attachment (PA × SME) (β = 0.070, *p* < 0.05) have a significant effect. This shows that social media usage for environmental information acquisition has a moderating function between place attachment and pro-environmental behavior, and model 6 also obtains the highest adjusted R-square value (0.285). This result proves H3. To answer H4, we need to compare the results of M2 and M4. M2 uses traditional media usage for environmental information acquisition (TME) as its independent variable. It can be found that the impact of traditional media usage for environmental information acquisition (TME) on pro-environmental behavior is not significant (β = 0.237, *p* > 0.05). M4 introduced social media usage for environmental information acquisition (SME) as an independent variable. It can be found that the impact of social media usage for environmental information acquisition (SME) is significant (β = 1.242, *p* < 0.001) and the impact of traditional media usage for environmental information acquisition (SME) is still not significant (β = −0.218, *p* > 0.05). This result proves H4. In other words, the correlation between traditional media and pro-environmental behavior is exactly lower than that between social media and pro-environmental behavior.

## 5. Conclusions and Discussion

This study mainly investigates social media’s impact on the relationship between place attachment and pro-environmental behavior. We discover that both place attachment and social media usage for environmental information acquisition have positive correlations with pro-environmental behavior. Social media moderates the relationship between place attachment and pro-environmental behavior. We also discover that traditional media usage for environmental information acquisition does not influence pro-environmental behavior as much as social media. This demonstrates social media’s importance in influencing pro-environmental behavior in the age of web 2.0. It provides insights into the specific relationship between people’s information acquisition and pro-environmental behaviors in a particular geographical context.

### 5.1. Theoretical Implications

#### 5.1.1. Social Media Take the Place of Traditional Media in Influencing Pro-Environmental Behaviors

Previous studies mainly examined media’s influence on pro-environmental behaviors from the perspectives of cultivation theory and use and gratifications theory, which usually focuses on changes in general behaviors and attitudes. For example, Chan [21] analyzed the impact of mass media as a subjective normative source on pro-environmental behavior. Ho. et al. [25] combined media dependence theory with planned behavior theory to analyze the impact of traditional media concerns, interpersonal communication, and media dependency on green buying behaviors and environmental participation actions. Huang [23] differentiated the specific influence of television, newspapers and the Internet on pro-environmental behaviors respectively. Similarly, Lee [22] also analyzed and compared the different effects of television, radio, Internet, newspapers, advertising, and interpersonal communication on pro-environmental behaviors. But few studies have focused on the role of social media in promoting pro-environmental behaviors. The only relevant research only suggests the possible models in which social media may influence pro-environmental behaviors. For example, Niki and Wilson [26] proved that social media’s comparison and display capabilities can promote pro-environmental behaviors. However, no research has been done on the different roles of traditional media and social media in influencing pro-environmental behaviors. Our research proves that social media is more important than traditional media in promoting pro-environmental behaviors.

#### 5.1.2. Social Media is Moderating Place Attachment’s Relations With Pro-Environmental Behaviors

Previous studies, including Halpenny [7], Scannell [11], Ramkissoon [18] and others, have repeatedly shown that place attachment does have a positive impact on pro-environmental behaviors. These studies have made people aware of the importance of relationship between humans and lands in pro-environmental behaviors. They also demonstrate the important role that emotions play in pro-environmental behaviors. Nevertheless, they have not noticed the specific ways in which place attachment functions in the era of social media. In fact, as Castells [37] has said, after the rise of the network society, local space and flow space have been reorganized, and people can simultaneously manage local connections in cyberspace. Especially when these connections combine interpersonal communication with media communication, people’s social space can be replicated in cyberspace. Our research shows that in the context of the current media environment, when we examine the influence of place attachment on pro-environmental behavior, we must consider the important role that social media plays. Without understanding the role of social media in moderating them, it will not be able to stimulate people’s place attachment and thus promote pro-environmental behaviors. This is crucial for us to understand the patterns of social behavior changes in the social media era.

### 5.2. Practical Implications

#### 5.2.1. Dissemination of Environmentally Relevant Information on Social Media Is a Very Effective Way to Promote Pro-Environmental Behaviors

This study proves that social media is playing a more important role than traditional media in promoting pro-environmental behaviors. The promotion and dissemination of various types of pro-environmental information on social media will directly contribute to the promotion of people’s pro-environmental behaviors, whereas the dissemination of various types of information on mass media cannot exert such influences. Social media’s promotion of pro-environmental behaviors may come from social comparison, social pressure, or because people are more likely to get a sense of self-efficacy in environmental protection. In short, as far as traditional media is concerned, our Chinese sample shows that disseminating pro-environmental information through social media is more effective in promoting pro-environmental behavior.

#### 5.2.2. Inspiring People’s Place Attachment through Social Media Is a Useful Way to Promote Pro-Environmental Behaviors

The research results show that place attachment is still exerting influence on pro-environmental behaviors, which fully proves the effect of man–land relationship in promoting local pro-environmental behaviors. But it must be noted that with the advent of the social media era that combines media communication with interpersonal communication, place attachment exerts its influence largely through the moderating function of social media. Therefore, if more information about place attachment and environmental awareness is provided by social media, it will be more conducive to promoting pro-environmental behaviors in countries like China. The premise of promoting pro-environmental behavior is that we must realize that people today are living on social media, and it is important to understand this.

### 5.3. Limitations

Because this research focuses on the mode in which place attachment affects pro-environmental behaviors in the social media era, the variables representing general demographic characteristics and the variables related to environmental protection are selected on the control variables, and traditional media usage for environmental information acquisition is introduced. It must be stated that the selection of these variables is only based on research design and selection of research intentions. In fact, there is no discussion on the role of planned behavior theory and normative behavior theory in explaining pro-environmental behaviors. How to organically integrate different research frameworks in our studies is the future direction of this research. At the same time, while this study compares the difference between traditional media and social media usage for environmental information acquisition in promoting pro-environmental behaviors, the role of interpersonal communication is not fully discussed. In addition, the specific mechanism by which social media and place attachment combine to affect pro-environmental behaviors has not been explored. These are all subjects for further research and expansion.

In terms of research method, this study only uses traditional regression analysis to compare the different models. If we want to examine how different dimensions of place attachment and social media influence pro-environmental behaviors, our analysis of structural equations in the future needs to be more appropriate and necessary. In addition, the study is based on the Chinese sample. Whether this relationship will change in other countries requires more data analysis and comparison of different national samples.

In any case, this study proves that in the social media era, the influence mechanism of place attachment on pro-environmental behavior is changing, and social media plays a more important role in changing people’s daily behavior. Social media is likely restructuring our model of cognition in a comprehensive manner. In this sense, we should firmly situate our research in the media environment of the WEB 2.0 era in order to understand people’s daily life and behaviors.

## Figures and Tables

**Table 1:** Distribution of sample socio-demographics.

	Categories	Frequency	Percentage (%)
Gender	Male	246	44.7
	Female	314	55.3
Education	Junior high school and below High school	6 15	1.1 1.7
	College/University	461	83.8
	Master	64	11.6
	Doctor and above	4	0.7
Income per year (Rmb)	<10,000	58	10.5
	10,000–30,000	49	8.9
	30,000–50,000 50,000–100,000	48 186	8.7 33.8
	100,000–200,000	168	30.5
	200,000–500,000	34	6.2
	>500,000	7	1.3
Age	Mean	30.5	

**Table 2:** Means, standard deviations, and inter-correlations of measurement.

	1	2	3	4	5	6	7	8	9	10	11	12
Gender												
Age	0.199 **											
Education	−0.025	−0.066										
Income	0.144 **	0.404 **	0.192 **									
CP	0.030	0.042	−0.022	0.122 **								
EK	−0.020	−0.122 **	0.176 **	−0.040	−0.118 **							
EC	−0.030	0.028	0.109 *	0.047	−0.136 **	0.300 **						
ERP	−0.068	0.070	0.043	0.029	−0.039	0.223 **	0.378 **					
TME	0.024	0.010	0.045	−0.072	0.097 *	0.133 **	0.199 **	0.115 **				
SME	0.041	−0.075	0.022	−0.039	0.220 **	−0.011	0.063	0.053	0.498 **			
PA	0.026	−0.002	0.037	0.039	0.221 **	0.054	0.220 **	0.189 **	0.215 **	0.216 **		
PEB	0.014	0.152 **	0.042	0.172 **	0.409 **	−0.116 **	0.003	0.152 **	0.070	0.235 **	0.289 **	
Mean	0.447	30.53	3.08	3.89	7.89	9.30	11.93	3.74	1.80	1.04	24.2	13.51
SD	0.498	7.68	0.46	1.43	3.11	1.23	3.76	0.53	0.80	0.60	6.64	3.89

Notes: * *p* < 0.05, ** *p* < 0.01,

**Table 3:** Multiple regression analysis for pro-environmental behavior (standard coefficient).

		M1	M2	M3	M4	M5	M6
	Gender	−0.011 (−0.360)	−0.109 (−0.361)	−0.167 (−0.567)	−0.178 (−0.598)	−0.225 (−0.773)	−0.023 (−0.677)
	Age	0.005 * (2.051)	0.045 * (2.089)	0.050 * (2.359)	0.056 * (2.614)	0.059 * (2.826)	0.057 ** (2.719)
	Education	0.046 (1.325)	0.612 (1.823)	0.596 (1.821)	0.600 (1.818)	0.587 (1.817)	0.584 (1.812)
	Income	0.020 (1.631)	0.192 (1.642)	0.182 (1.589)	0.188 (1.633)	0.179 (1.584)	0.171 (1.523)
	CP	0.050 *** (10.206)	0.484 *** (9.970)	0.423 *** (8.681)	0.443 *** (8.017)	0.391 *** (8.017)	0.397 *** (8.152)
	EK	−0.035 ** (−2.645)	−0.369 ** (−2.856)	−0.352 ** (−2.790)	−0.322 * (−2.525)	−0.311 * (−2.490)	−0.304 * (−2.441)
	EC	0.001 (0.245)	0.027 (0.601)	−0.013 (−0.306)	0.026 (0.594)	−0.011 (−0.263)	−0.020 (−0.463)
	ERP	0.133 *** (4.263)	1.365 *** (4.491)	1.177 *** (3.941)	1.322 *** (4.422)	1.152 *** (3.909)	1.147 *** (3.902)
	TME		0.237 (1.248)	0.089 (0.473)	−0.218 (−1.020)	−0.311 (−1.481)	−0.310 (−1.481)
*Independent variable*	PA			0.123 *** (5.270)		0.114 *** (4.963)	0.051 (1.275)
*Moderating variable*	SME				1.242 *** (4.380)	1.119 *** (4.013)	2.730 ** (3.171)
*Interaction variable*	PA × SME						0.070 * (1.977)
	F	19.974 ***	18.657 ***	20.401 ***	19.275 ***	20.530 ***	19.247 ***
	AdjustedR^2^	0.217	0.224	0.261	0.250	0.281	0.285

Notes: * *p* < 0.05, ** *p* < 0.01, *** *p* < 0.001.
